# Determination of the Minimum Cell-to-Cell Adhesion Time Using Optical Tweezers in Leukemia and Lymphoma Research

**DOI:** 10.3390/mps8030059

**Published:** 2025-06-04

**Authors:** Kamila Duś-Szachniewicz, Sławomir Drobczyński

**Affiliations:** 1 Department of Clinical and Experimental Pathology, Institute of General and Experimental Pathology, Wrocław Medical University, 50-368 Wrocław, Poland; 2 Department of Optics and Photonics, Faculty of Fundamental Problems of Technology, Wrocław University of Science and Technology, 50-370 Wrocław, Poland; slawomir.drobczynski@pwr.edu.pl

**Keywords:** optical tweezers, leukemia–lymphoma (LL) cell lines, minimum cell-to-cell adhesion time, single cell adhesion assay, hematological malignancies

## Abstract

Single-cell adhesion assays can be divided into studies on attachment and detachment events, and several methods that enable the characterization of both processes have been established in the past. Due to their low invasiveness, label-free principles, and contactless operation, optical methods are especially beneficial for this purpose. Historically, optical tweezers (OTs) have been used to explore single-cell detachment events, allowing for the precise determination of minute physical forces. However, it has been noted that OTs can also be used to study single-cell attachment dynamics, including the evaluation of minimum cell-to-cell contact times necessary to establish a stable adhesive bond. Here, we provide a step-by-step protocol to effectively evaluate minute changes in the adhesion of single leukemia–lymphoma cells using optical tweezers with low laser intensities. This serves as a valuable in vitro model to determine the effects of physical and chemical factors on the adhesive properties of leukemia–lymphoma (LL) cells.

## 1. Introduction

Cell adhesion refers to the ability of cells to form direct contacts with other cells (cell-to-cell adhesion) or with the extracellular matrix (cell-to-ECM adhesion), providing structural integrity to each multicellular organism [1,2]. The complex process of cell-to-cell adhesion involves the recognition and binding of membrane proteins, collectively called cell adhesion molecules, as well as the generation and transmission of mechanical forces [3,4].

Importantly, the adhesive properties of human cells are closely related to health status and function; changes in cell adhesion underline a wide range of diseases, including cancers [5,6,7]. Generally, cancer cells exhibit reduced adhesiveness compared to their corresponding healthy cells [8,9,10], which results in the destruction of normal histological structures. Moreover, changes in the adhesion of cancer cells to the extracellular matrix are related to the acquisition of the potential for invasion and metastasis [11,12]. It was recently found that the overall adhesion strength of a single cancer cell is approximately constant in all phases except the mitotic phase, where it is significantly lower. This suggests that single-cell adhesion is a promising target for selective cancer drugs [13]; however, the development of new sophisticated methods is required.

In this work, we present a method for the evaluation of cell-to-cell adhesion, developed based on the interactions between white blood cells (normal or pathological) and their microenvironment, represented by bone marrow stromal cells. In fact, both leukemia and lymphoma cells interact with stromal cells (SCs) in the bone marrow (BM) via direct cell contact, and this interaction has been shown to be a leading contributor to chemoresistance in hematological patients [14,15,16,17]. The model proposed here permits the controlled assembly of in vitro interactions that mimic the circuits found naturally in the bone marrow environment.

Several methods for characterizing cell adhesion have been established in the past. Despite significant progress in our understanding of cellular adhesion processes in health and disease, numerous areas still require exploration. In general, cell adhesion studies can focus on single cells as well as on populations of cells, and in both of these categories, cell attachment or detachment mechanisms can be analyzed [2]. The most widely used techniques in laboratories include bulk assays (e.g., washing assays, spinning disk methods, or microfluidics) based on hydrodynamic shear flow that removes cells from the surface and represents population-level behavior [18]. While bulk assays are easy, inexpensive, and effective for the rapid production of statistically relevant data, they do not provide insights into single-cell behavior. Meanwhile, the manipulation of single cells provides essential information on how individual cells interact with each other and their microenvironment in health and disease [19,20,21,22]. The pursuit of increasingly precise research techniques can facilitate new discoveries in collective cancer progression that may be targeted for cancer therapy. From a technical point of view, precision in determining the adhesive properties of individual cells is unattainable using standard methods, which may result in the inability to detect small changes in adhesion that could have potential biological significance [21]. For instance, the time required for initial cell-to-cell adhesion to occur in population studies is typically over 30 min. In contrast, using single-cell adhesion assays, we found that the formation of initial adhesion is much more rapid, ranging from 10 to 360 s for a variety of tested lymphoma and leukemia cell lines [23,24,25,26].

Optical tweezers (OTs) are an innovative tool that utilize a beam of laser light, focused through a microscope objective lens, for the micromanipulation of nano- and micro-objects and particles without mechanical contact [27,28,29]. The experimental setup of OTs primarily consists of mirrors and lenses for beam focusing and steering; however, it can vary from a very simple single-beam setup to a highly complex multibeam holographic system [30,31]. Today, commercial systems are becoming increasingly popular, providing the opportunity to trap, move, and apply calibrated forces to microscopic refractive objects for a wide range of applications in research [28]. Among other fields, optical tweezer technology is having a growing impact in the biomedical field, particularly in basic studies of molecular and cellular biology [32,33,34]. One of the areas being explored is cell adhesion research [35,36]. Historically, OTs have been used to explore single-cell detachment events, focusing on the precise determination of adhesion force values in the pico-Newton scale [37,38,39]. However, it has been noted that OTs can also be used to study single-cell attachment dynamics, including the evaluation of cell–substrate contact times [38,39,40]. We have previously employed optical trapping to conduct precise measurements of the dynamics of minimum cell-to-cell adhesion time [23,24,25,26]. Based on the average minimum adhesion time values of LL cells to bone marrow stromal cells, we reported that individual LL cell lines significantly differ in their adhesive properties [23]. Next, by evaluating the minimum cell-to-cell adhesion time, we also demonstrated extensive changes in LL cell adhesion after drug and hypoxic treatment [24,25]. Furthermore, we reported a significant decrease in the average minimum adhesion time in lymphoma cells compared to healthy lymphocytes and described how to discriminate individual lymphoma cells from normal lymphocytes using optical tweezers [26].

In this paper, we present an optimized, efficient, and reproducible method for the precise measurement of minimum cell-to-cell adhesion time using laser tweezers. This assay can be utilized with both custom-built systems and commercially available optical tweezers, and the basic steps for cell preparation, system calibration, and optical trapping remain the same.

## 2. Experimental Design

**Figure 1:**
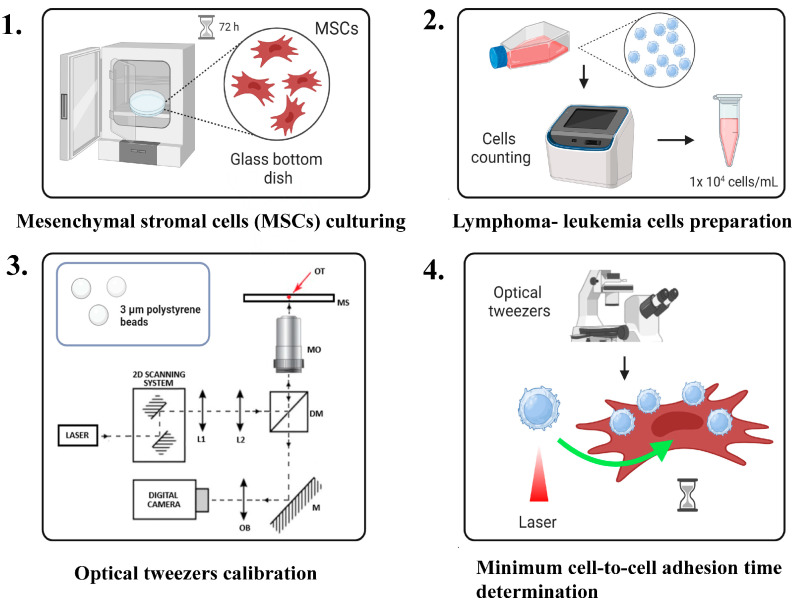
Summary of the evaluation of the minimum cell-to-cell adhesion time using optical tweezers (OTs). This image was created with BioRender.com (accessed on 25 March 2025).

The minimum cell-to-cell adhesion time was measured at the single-cell level using optical tweezers. The basic steps involved culturing and preparing lymphoma and stromal cells for optical trapping, calibrating and setting up the optical tweezers, and evaluating the minimum cell-to-cell adhesion time as a lymphoma cell is trapped and brought into contact with a stromal cell. A visual scheme illustrating the sequence of protocol steps is presented in Figure 1 (please also see Videos S1 and S2 in the Supplementary Materials). Next, we optimized our protocol to characterize minute changes in single-cell adhesion as a consequence of hypoxia (Downstream Application 1) and drug treatment (Downstream Application 2).

**Figure 2:**
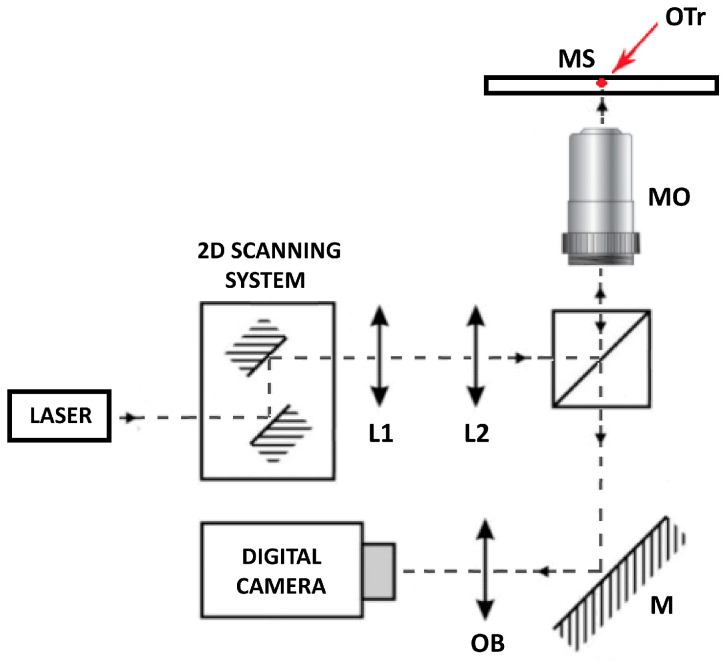
Schematic drawing of the optical tweezers (OTs) setup: a single-mode Nd:YVO4 laser focused by Olympus UplanFLN 100×/1.3 microscope objective provide trapping efficiency with minimal cell photodamage effect. DM: dichroic mirror; L1, L2: lenses; M: mirror; OB: objective; MO: microscope objective; OTr: optical trap; MS: microscopic sample.

The OT system used in this study is based on the Olympus IX71 inverted biological microscope with laser beam introduced through the rear port, as shown in Figure 2. Maximum 1064 nm laser power is 4 W limited by the Thorlabs VA5-1064 polarization attenuator. The laser power was measured using a Thorlabs PM100 USB power meter with an S121C detector. The detector was placed in the position of the microscope objective. Power measurement in the sample plane is somewhat challenging as it requires immersion between the objective and the detector; a focused laser beam at the diffraction limit can damage the detector in this setup. By knowing the input power and the transmittance of the microscope objective for the 1064 nm laser, we estimated the power at the sample plane.

The sample was illuminated by the original Olympus IX71 microscope illumination system. For the required measurements of trap stiffness, a fast Microtron MC1362 camera and the spectrum analysis method were used [41]. Precise movement of the laser beam was obtained with Thorlabs GVS002 XY galvo-scanners, and macro displacements within the sample were performed manually using the x–y stage of the Olympus IX71 microscope.

### 2.1. Reagents

1. RPMI 1640 Medium (Gibco, Waltham, MA, USA, Cat. No.: 11875093).
2. 1% Penicillin/Streptomycin (Gibco, Waltham, MA, USA, Cat. No.: 15140122).
3. Fetal Bovine Serum (FBS) (Gibco, Waltham, MA, USA, Cat. No.: A5256701).
4. HEPES buffer (Sigma-Aldrich, Darmstadt, Germany, Cat. No.: H4034-25G).
5. Phosphate-buffered saline (PBS), pH 7.4 suitable for cell culture (Gibco, Waltham, MA, USA, Cat. No.: 10010023).
6. Trypsin-EDTA, 0.05% (Gibco, Waltham, MA, USA, Cat. No.: 25300096)
7. Trypan blue (TB) solution, 0.4% (Gibco, Waltham, MA, USA, Cat. No.: 15250061).
8. Sonidegib (NVP-LDE225) (Selleck Chemicals, Houston, TX, USA, Cat. No.: S2151).
9. Doxorubicin (Sigma-Aldrich, Darmstadt, Germany, Cat No.: D1515).
10. AMD3100 (plerixafor), (Sigma-Aldrich, Darmstadt, Germany, Cat No.: 239820).
11. Dimethyl sulfoxide (DMSO) for molecular biology (Sigma-Aldrich, Darmstadt, Germany, Cat. No.: D8418).
12. Sterile water.

### 2.2. Materials

1. Pipette 100–1000 µL (Eppendorf, Hamburg, Germany, Cat. No.: 3123000063).
2. Pipette 20–200 µL (Eppendorf, Hamburg, Germany, Cat. No.: 3123000055).
3. Pipette 2–20 µL (Eppendorf, Hamburg, Germany, Cat. No.: 3123000098).
4. Pipette filter tips: 10 uL, 100 µL, 1000 µL (VWR, Radnor, PA, USA, Cat. No.: 613-5091; 613-0860, and 613-0899).
5. Sterile serological pipettes: 10 mL (Grenier Bio-One, Frickenhausen, Germany, Cat. No.: 607180).
6. Eppendorf safe-lock tubes: 1.5 mL (Eppendorf, Hamburg, Germany, Cat. No.: 0030120086).
7. T25 flasks for cell suspension (Sarstedt, Nümbrecht, Germany, Cat. No.: 83.3910.502).
8. T75 cell culture treated flasks (Sarstedt, Nümbrecht, Germany, Cat. No.: 83.3911).
9. Centrifuge tubes: 15 mL (Eppendorf, Hamburg, Germany, Cat. No.: EP0030122194).
10. Sterile high glass-bottom 35 mm dish (IBIDI, Martinsried, Germany, Cat. No.: 81158).
11. Petri Dish, PS, 150 mm × 20 mm, 148 cm^2^, sterile (SPL Life Science, Suwon, Republic of Korea, Cat. No.: S10150).
12. EVE Cell Counting Slides (NanoEntek, Seoul, Korea, Cat. No.: NE-EVS-50).

### 2.3. Cell Lines

1. Ri-1 (B-cell non-Hodgkin lymphoma, German Collection of Microorganisms and Cell Cultures (DSMZ), Braunschweig, Germany, Cat. No.: ACC 585).
2. RAJI (Burkitt lymphoma, American Type Culture Collection (ATCC), Manassas, VA, USA, Cat. No.: CCL-86).
3. Toledo (B-cell non-Hodgkin lymphoma, ATCC, Manassas, VA, USA, Cat. No.: CRL-2631).
4. OCI-AML3 (acute myeloid leukemia, DSMS, Braunschweig, Germany, Cat. No.: ACC 582).
5. H-5 (mesenchymal stromal cells, ATCC, Manassas, VA, USA, Cat. No.: CRL-3611).

### 2.4. Equipment

- Centrifuge for cell culture (EBA 20, Hettich Zentrigugen, Tuttlingen, Germany; Cat. No.: 2002).
- Inverted microscope for cell culture (Olympus, Tokyo, Japan, Cat. No.: IX73).
- Standard 37 °C, 5% CO_2_ humidified cell culture incubator (Esco Technologies, Inc., Saint Louis, MO, USA, Cat. No.: CCL).
- Multigas incubator (Galaxy 48 R/48 S CO_2_, Eppendorf, Hamburg, Germany; Cat. No.: CO48332001).
- Biological safety cabinet (Scanlaf, Höör, Sweeden, Cat. No.: 9.002.022.000).
- Automated Cell Counter (NanoEntek, Seoul, Republic of Korea; Cat. No.: EVE-MC)
- Vortex (NeoLab, Heidelberg, Germany, Cat. No.: D-6012).

### 2.5. Optical Tweezers Setup

- Inverted microscope with an UplanFLN 100×/1.3 microscope objective. (Olympus, Hamburg, Germany, Cat. No.: IX71).
- Nd:YAG 1064 nm laser (Laser Quantum, Stockport, UK, Cat. No.: Ventus 1064).
- Galvano-mirror XY scanning system (Thorlabs, Newton, NJ, USA, Cat. No.: GVS002).
- Lense 1 (Thorlabs, Newton, NJ, USA, Cat. No.: AC254-050-B-ML).
- Lense 2 (Thorlabs, Newton, NJ, USA, Cat. No.: AC508-200-B-ML).
- Dichroic mirrors (Thorlabs, Newton, NJ, USA Cat. No.: DMSP805R).
- Digital camera (Mikrotron GmbH, Unterschleißheim, Germany, Cat. No.: MC1362).

## 3. Procedure

### 3.1. Mesenchymal Stromal Cell Line Maintenance and Preparation for Experiments

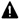

**CRITICAL STEP 1:** Start by preparing the glass-bottom dishes with mesenchymal stromal cells from the H-5 cell line for 72 h prior to manipulations using optical tweezers. For a single experiment, approximately 5 dishes will be required. H-5 cells were purchased from American Type Culture Collection (ATCC) and stored in liquid nitrogen until use. We recommend using early-passage cells (up to passage 10), along with associated histological and genetic analyses.

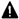

**CRITICAL STEP 2:** Prepare RPMI medium supplemented with 10% FBS, 1% Penicillin/Streptomycin, and 10 mM HEPES buffer. The required volumes should be incubated in a water bath at 37 °C prior to use in cell culture.

1. Culture the mesenchymal stromal cells of the H-5 line aseptically in T75 flasks under standard conditions (37 °C, 5% CO_2_) in complete RPMI 1640 medium.
2. When the cells reach 80–90% confluence, proceed to enzymatic dissociation.
3. Aspirate the cell medium and wash the cell monolayer twice with pre-warmed phosphate-buffered saline (PBS).
4. Add 2 mL of 0.05% trypsin-1 mM EDTA to cover the cells and incubate the flask at 37 °C for 5 min to facilitate cell detachment.
5. Add 2 mL of pre-warmed complete RPMI-1640 medium to stop the action of trypsin.
6. Gently suspend the H-5 cells with a 10 mL serological pipette, collect the cells, and transfer them into a sterile 15 mL tube.
7. Centrifuge the cells at 300× *g* for 7 min, discard the supernatant, and resuspend the cell pellet in 1 mL of pre-warmed culture medium.
8. Count the number and viability of stromal cells, for example, using an automated cell counter and Trypan blue (TB) dye.
9. Dilute the H-5 cells to the desired concentration of 2 × 10^5^ cells/mL.
10. Seed 100 µL of the cell solution as a single drop in the central part of the glass-bottom dish.
11. Place the glass-bottom dishes in a large Petri dish. To prevent the cells from drying out, add an extra dish with sterile PBS.
12. Gently transfer the dishes to the incubator, avoiding spilling the drop to the sides.
13. After 24 h of incubation, wash off the cells that have not adhered to the glass with 2 mL of PBS, and add 1 mL of fresh pre-warmed culture medium.
14. Incubate the cells for the next 48–72 h until they reach a spindle-shaped morphology.

### 3.2. Culturing and Preparation of Leukemia/Lymphoma (LL) Cell Lines for Experiments

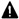

**CRITICAL STEP:** Leukemia–lymphoma cell lines are cultured in RPMI 1640 medium containing FBS and antibiotics according to the manufacturer’s instructions. Low-passage-number samples need to be generated for use in subsequent experiments for each cell line, frozen, and stored in liquid nitrogen until needed.

1. Thaw the vial by gentle agitation in a 37 °C water bath.
2. Seed the LL cell lines in complete culture medium in T25 flasks dedicated to cell suspension. Use the seeding densities presented in Table 1.
3. Maintain the cell culture at the optimal cell density (please see Table 1) by splitting the saturated culture 1:2 to 1:3 every 2–3 days.
4. Prior to preparing LL cell suspensions for the experiments, transfer the contents of the flask to a centrifuge tube containing 9 mL of complete culture medium and centrifuge the cells at 300× *g* for 7 min at room temperature.

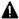

**CRITICAL STEP:** Start preparing samples no earlier than 30 min before the planned experiment.

5. Discard the supernatant and resuspend the cell pellet with 4 mL of complete culture medium.
6. Determine the cell viability with Trypan blue, which should be >95%.
7. Prepare the cell suspension at a concentration of 1 × 10^4^ cells/mL.
8. Incubate the sample at 37 °C until optical manipulations.

### 3.3. Optical Tweezer Experimental Setup and Calibration

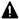

**CRITICAL STEP:** In this work, we do not focus on the optical tweezer building protocol; rather, we provide the main functional steps of optical tweezer systems, which are achieved in both commercial and custom-built systems. In our custom-built system, presented in Figure 2, the given trap stiffness was achieved for a laser power of 100 mW at the entrance of the microscope objective, measured with a 3 µm polystyrene bead (Polybead Microspheres, Polysciences, Warrington, PA, USA). The minimal invasiveness of the laser on lymphoma cells was previously confirmed [23,24,25,26] while still maintaining the ability to manipulate living cells. All procedures were carried out at room temperature, specifically 22–24 °C.

1. Turn on the laser and adjust the power to achieve a trap stiffness of approximately 50 pN/µm in the optical tweezer system.
2. Allow the laser to stabilize by keeping it on for about 10 min before beginning any work.
3. Launch the computer software. In the software window, you will see an image of the microscope sample. Utilize the markers displayed on the screen to move the optical trap in any desired direction.

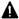

**CRITICAL STEP:** In our study, we used custom software written in Visual Studio 2019 C++. The program displays an image of a microscopic sample on the monitor and couples the mouse cursor movements with the control of the Thorlabs GVS002 XY galvo-scanners, enabling precise movement of the focused laser beam to the locations indicated by the cursor on the screen.

4. Add 30 μL of immersion medium to the center of the microscope objective and place a 35 mm glass-bottom microscopy dish on the microscope stage.
5. Carefully turn the micrometer adjustment screw of the microscope up and down until the laser beam is sharply focused into a single, bright point on the screen.
6. Add 1 × 10^3^ LL cells to 100 µL of culture medium and place the cell suspension in the center of the glass-bottom dish as a single drop. Wait for approximately 5 min until the cells sink to the bottom of the dish.
7. Use the microscope stage movements to locate a floating lymphoma cell. Activate the optical trap by clicking on the trap cursor on the screen to engage with the cell. Attempt to move the LL cell to the desired position. A cell suitable for manipulation must be able to move easily in the intended direction.

### 3.4. Trapping Procedure and Determination of Minimum Cell-to-Cell Adhesion

**Figure 3:**
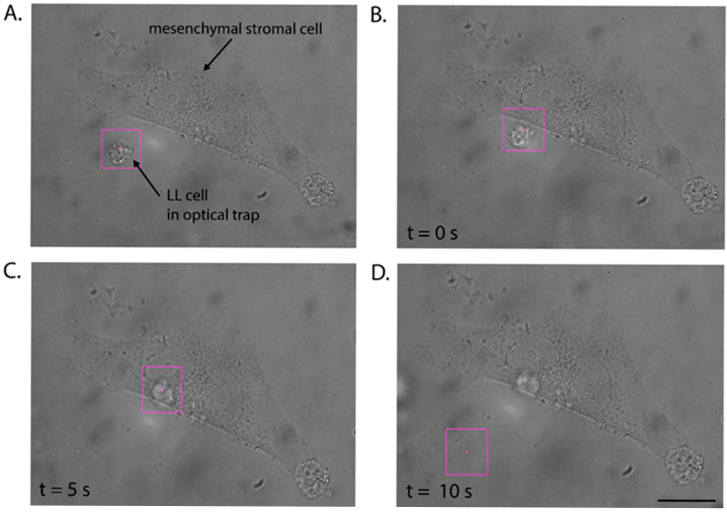
Determination of the minimum cell-to-cell adhesion time using optical tweezers (OTs). (**A**) The single leukemia–lymphoma (LL) cell is optically trapped and assessed for its ability to move freely. (**B**) The LL cell is relocated toward the membrane of the mesenchymal stromal cell (MSC) growing on the glass-bottom dish. (**C**) The cell is placed on the central region of the MSC and kept in this position for 5 s to initiate cell–cell adhesion. (**D**) The optical trap is moved approximately 25 μm away from the interacting cells for 10 s, followed by three detachment attempts. The scale bar is 20 μm.

1. Place a glass-bottom dish with mesenchymal stromal cells on the microscope stage.
2. Position the MSC cell, to which the LL cell will be attached, in the field of view of the microscope.
3. Add 100 µL of the previously prepared suspension of LL cells. Wait approximately 5 min until the cells sink to the bottom.
4. Identify a candidate cell under the microscope and position the optical trap over the cell of interest.
5. Trap the cell with the optical tweezers (Figure 3A) and attempt to move it. The optical trap should allow the cells to move freely in the direction specified by the operator.
6. Gently bring the LL cell into contact with the central part of the membrane of the MSC cell (see Figure 3B) and hold the cell in this position with the optical trap for 5 s (see Video S1, Figure 3C).
7. Next, move the optical trap approximately 25 μm away from the interacting cells for 10 s (see Video S1, Figure 3D).
8. Check if the given contact time is sufficient to initiate the formation of the adhesion junction between the cells. For this purpose, catch the LL cell in the optical trap and attempt to pull it away from the stromal cell during three detachment attempts (see Video S2).
9. If the optical trap with the LL cell is retracted from the surface of the stromal cell, extend the adhesion time to 10 s.
10. Repeat steps 7 and 8 to check if the nascent adhesion has formed. After an unsuccessful attempt, increase the contact time between the cells.
11. Once the LL cell is permanently attached to the stromal cell, note the time at which adhesion occurred, and repeat the procedure with the next LL cell.

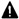

**CRITICAL STEP:** To increase the throughput of the procedure, several LL cells can be attached to one MSCs cell, but LL cells should not adhere to each other. A glass-bottom dish with MSCs cells should be changed every 20 min.

**TROUBLESHOOTING TIPS:**

1. It is recommended to set the initial contact time as 5, 10, 15, 20, 30, 40, 60, 90, 120, 150, 180, 210, 240, 270, 300, and 360 s [23,26].
2. The mean adhesion time will depend on the cell type and state. In general, distinct LL cell lines and primary cells differ significantly in average minimum adhesion time, ranging from 15.5 ± 8.4 to 132.9 ± 48.8 s for untreated B-cell lymphoma cell lines [23,24]. Thus, it is necessary to determine time intervals for each cell line in a preliminary experiment involving approximately 10 individual cells and adapt these values to the adhesive properties of the examined cells. For example, if the average minimum cell-to-cell adhesion time is established as 120 s, one can set the initial contact time to 60 s and then increase it successively every 30 s until a stable adhesive bond is formed.
3. A single B-cell should be assembled to the stromal cell a maximum of three times, and the entire time of individual cell manipulation should not exceed 420 s due to the risk of photodamage. This aspect was carefully addressed in our previous works [23,24,25,26].
4. To avoid interferences with other cells, we used a cell concentration of 1 × 10^5^ LL cells/mL and discarded measurements with more than one cell inside the microscope field.
5. MSCs used for experiments may exhibit specific shape geometries; however, the lymphoma cell should be positioned over a central region of the mesenchymal stromal cell near the nucleus.
6. Control the lymphoma cell morphology during the experiment. If you observe an irregular bulge in the plasma membrane (blebbing of the plasma membrane), stop the experiment and apply a new dish with stromal cells.

### 3.5. Downstream Application 1: The Changes in Adhesion Under Hypoxic Treatment

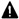

**CRITICAL STEP 1:** Cells can enter a state of hypoxia at varying oxygen concentrations; so, this should be investigated beforehand, for example, by evaluating the status of hypoxia-inducible factor 1α (HIF-1α). In our previous study, a significant increase in HIF-1α activity was detected in cells growing in the presence of 1% oxygen for 24 h; therefore, these conditions were considered hypoxic.

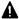

**CRITICAL STEP 2:** For the evaluation of cell adhesion evaluation in hypoxia, a concentration of 1% oxygen was maintained during the manipulations by using an in-house fabricated hypoxic chamber [23].

1. Culture LL cell lines as previously described in Section 3.2.
2. Prior to hypoxic treatment, centrifuge, count, and seed cells in a new T25 flask according to the densities presented in Table 1. Prepare two identical flasks (twin cultures) for each cell line.
3. In parallel, prepare MSCs as presented in Section 3.1.
4. Set a multigas incubator to 1% oxygen (1% O_2_, 5% CO_2_, 94% N_2_).
5. Preincubate the complete culture media in 1% oxygen overnight to reduce the O_2_ contained in the media.
6. Place the flasks with LL cells and MSCs in hypoxia for 24 h.
7. As a control, cultivate a T25 flask with the corresponding cells in complete RPMI in parallel for the same duration under standard (normoxic) conditions in a conventional incubator.
8. After 24 h of cell cultivation under normoxia and hypoxia, centrifuge the cells, resuspend the cell pellet in 4 mL of culture medium, and determine the cell number.
9. Homogenize the cell pellet quickly by pipetting up and down. Count the cells and prepare a suspension of 1 × 10^4^ cells/mL.
10. Mount the hypoxic chamber on the motorized stage of the microscope. Control the level of oxygen inside the chamber.
11. Place the glass-bottom dish with stromal cells inside the hypoxic chamber.
12. Proceed with the manipulation with the optical tweezers as described in Section 3.4.

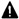

**CRITICAL STEP:** The initial contact time required to establish cell-to-cell adhesion must be experimentally determined for each cell line during preliminary studies.

### 3.6. Downstream Application 2: The Changes in Adhesion Under Drug Treatment

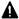

**CRITICAL STEP:** The optimal time and dose of doxorubicin (DOX) and plerixafor (AMD3100) on Raji cell lines were previously investigated [24,42]. However, each LL cell line should be tested at various concentrations of drugs and incubation times in order to optimize the protocol.

1. Prepare a stock solution of DOX (1 mM) and AMD3100 (5 mM) by dissolving the drugs in sterile deionized water. Store at −20 °C until use.
2. Culture the Raji cell line as previously described in Section 3.2.
3. To test the influence of the drugs on single cell adhesion, seed 5 × 10^5^ Raji cells in 1 mL of culture medium in a 12-well plate.
4. Add either DOX, AMD3100, or both drugs to the Raji cells, resulting in final concentrations of 0.1 μM for DOX and 50 μM for AMD3100 in the culture medium.
5. Incubate the cells under standard conditions for 48 h.
6. Centrifuge the cells at 300× *g* for 7 min, remove all liquid, and add 4 mL of PBS; then, vortex to wash the cells.
7. Centrifuge the cells again at 300× *g* for 7 min and remove all liquid using a serological pipette.
8. Resuspend the cell pellets by adding 4 mL of fresh culture medium, count the cells, and determine cell viability using the TB assay.
9. Prepare a working cell suspension of 1 × 10^4^ cells/mL.
10. Proceed with the manipulation with the optical tweezers as described in Section 3.4.

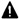

**CRITICAL STEP:** The initial contact time required to establish cell-to-cell adhesion must be experimentally determined for each cell line during preliminary studies.

## 4. Expected Results and Discussion

Distinguishing between various cells and cell states is crucial for disease identification, predicting patient outcomes, and evaluating drug responses [6]. Cell adhesion, among other factors, is an important feature in assessing cell health and disease status. In fact, two major hallmarks of cancer, loss of cell-to-cell adhesion, and anchorage-independent growth, are related to adhesion processes [7,43]. The adhesive properties of an individual cell can be determined by studying various cell–substrate contact times, both in population studies and at the single-cell level [40]. In our method, optical tweezers (OTs) are used as a tool for selectively capturing, transporting, and precisely targeting a single leukemia–lymphoma (LL) cell on a stromal cell, allowing for the controlled construction of a single-cell adhesion assay [23,24,25]. It should be noted that although we developed our method using custom-built OTs, it is also appropriate for commercially available systems. For practical reasons, we decided not to measure the trap force directly. Instead, we determined the laser power that allows us to move cells without exposing them to thermal damage. Previously, we reported precise trap force measurements for well-defined objects such as polystyrene beads attached to cell membranes [44]. However, such measurements are very time-consuming and complex. These experiences motivated us to search for a more practical approach. When moving relatively large objects such as cells, optical tweezers operate at the boundary between the external medium and the cell membrane. Since human LL cells vary in shape and size [45], the force exerted by the optical trap is also variable. Therefore, we performed multiple pre-measurements and averaged the obtained results.

Both leukemia and lymphoma are cancers that share a similar cellular origin. These cancers grow in vitro in cell suspension, resulting in an unchanged shape during cell culture. Leukemia–lymphoma (LL) cell lines serve as an important model system in the field of cancer research [46,47]. Owing to the comprehensive documentation of their oncogenomic and transcriptional changes, LL cell lines can be matched to their parent tumors with a level of accuracy that is not achievable in other types of cancer [47]. In our laboratory, we have employed various LL cell lines to create a single-cell adhesion assay.

To accomplish this, we measured the minimum cell-to-cell adhesion time between individual LL cells and mesenchymal stromal cells (MSCs) using optical tweezers. Figure 2 demonstrates a sequence of optical manipulation steps. In short, the LL cell is optically trapped (Figure 3A) and moved toward the membrane of the stromal cell (Figure 3B). Next, the LL cell is placed on the central part of the MSC and maintained in this position for a specified time interval to initiate cell–cell adhesion (Figure 3C), followed by optical testing to determine if the given contact time is sufficient to initiate the formation of the adhesion junction between the cells through three detachment attempts (Figure 3D). The details of the experimental procedure are further presented in Videos S1 and S2.

**Figure 4:**
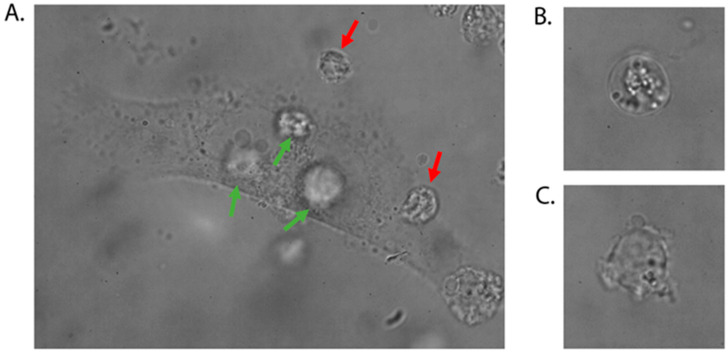
Details of the optical trapping procedure. (**A**) The leukemia–lymphoma (LL) cells indicated by green arrows are properly placed on the central part of the mesenchymal stromal cell (MSC) to initiate cell–cell adhesion. Note that LL cells do not stick to each other. The red arrows point to cells located too close to the edges of the MSCs, which prevents the maintenance of reproducible measurement conditions. (**B**) Proper morphology of the LL cell used for optical manipulation. (**C**) Cell exhibiting an irregular shape and physical changes in the plasma membrane (including protrusions and disrupted integrity).

The entire procedure is performed in a glass-bottom dish and the cells are selected by the operator based on their microscopic images. This technique does not require any chemical modification or preincubation (it is label-free), which guarantees the maintenance of conditions as close as possible to in vivo conditions. Our method offers the advantage of non-invasively individually analyzing the adhesive properties of numerous cells in a single experiment, all within a relatively brief timeframe, which varies according to the adhesive characteristics of each cell line. Based on our experience, a skilled operator can assess approximately 40–50 cells from highly adhesive cell lines within a 60 min session of optical manipulation [23,24,25,26]. To measure the minimum adhesion time even more effectively, the single MSCs may be used for evaluation of minimum cell–cell adhesion time for several LL cells. Figure 4A illustrates how individual LL cells should be positioned on the central part of the MSCs (where the nucleus is located) to: (1) maintain reproducible experimental conditions, and (2) avoid redundant interactions between LL cells. It is also essential to select cells for manipulation that have a regular circular shape and an intact cell membrane (Figure 4B). Cells with visible protrusions (blebs), irregular shapes, or physical changes in the plasma membrane, as seen in Figure 4C, are not suitable for this experiment.

**Figure 5:**
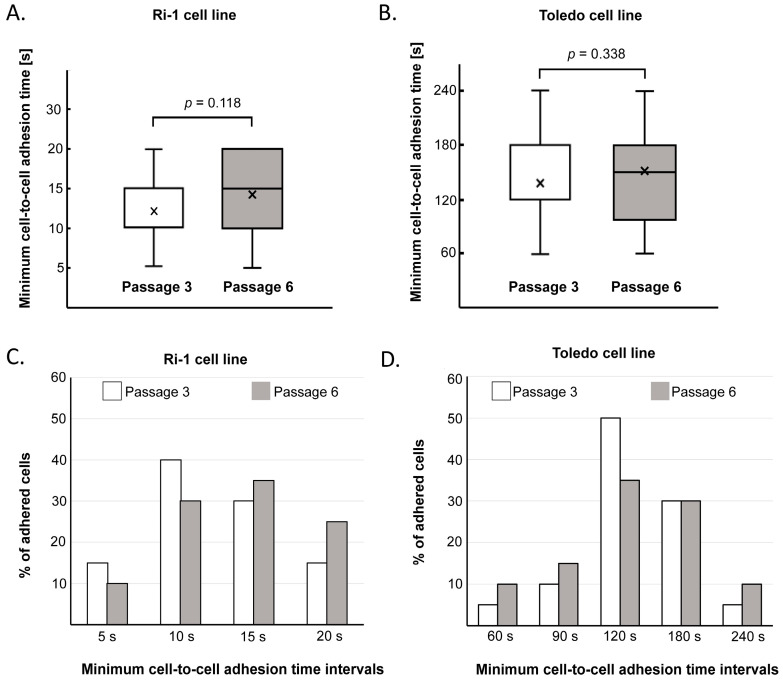
Minimum cell-to-cell adhesion time of leukemia–lymphoma (LL) cell lines evaluated in optical tweezers (OTs). The minimum adhesion time required for (**A**) the Ri-1 cell line and (**B**) the Toledo cell line to adhere to mesenchymal stromal cells (MSCs). The percentage distribution of (**C**) Ri-1 cells and (**D**) Toledo cells adhered to MSCs within distinct time intervals. Adhesion assessments were conducted for both passage 3 and passage 6 of each cell line. No statistically significant differences were observed between the two passages in either cell line. *N* = 20 for each experimental groups, Student’s *t*-test.

Our protocol, due to its precision and repeatability in measurement, is valuable for studying minute changes in cell adhesion, as shown in Figure 5. Initially, we observed substantial differences in the adhesive properties among the Ri-1 and Toledo cell lines representing B-cell non-Hodgkin lymphoma (B-NHL). Individual Ri-1 cells adhered to mesenchymal stromal cells within a time frame of 5 to 20 s (Figure 5A), while Toledo cells required a longer duration, with adhesion times ranging from 60 to 240 s (Figure 5B). Specifically, the average minimum cell-to-cell adhesion time for Ri-1 cells was 12.83 ± 4.94 s, which is nearly 11 times faster than the Toledo cell line’s average adhesion time of 141 ± 49.69 s. Figure 5C,D presents the percentage of Ri-1 and Toledo cells that adhered to MSCs across increasing time intervals. Importantly, adhesion assessments were performed for both passages 3 and 6 of each cell line, and no statistically significant differences were observed between the two measurements. The data presented indicate that the adhesion properties of individual LL cells are specific to their respective cell lines, consistent with the findings of our previous study [23]. This difference in adhesion between cell lines may suggest variations in the cell surface properties or the underlying mechanisms that regulate adhesion in LL cell lines. Such observations were facilitated by the use of optical tweezers, which provide a level of precision significantly greater than that attained with popular wash assay for measuring cell adhesion, where the minimum measurable time for adhesion to occur is typically no less than 30–60 min. Furthermore, traditional methods primarily focus on measuring the ratio of attached cells to non-attached cells within a specific time frame rather than the precise time of adherence. As a result, they often overlook the subtle nuances in intracellular molecular expression among individual cells, leading to the loss of crucial biological information [2,48]. In contrast, examining cells at the single-cell level provides a robust approach to exploring cell heterogeneity and greatly enhances our understanding of the behaviors of tissues, organs, and entire living organisms [49,50,51].

**Figure 6:**
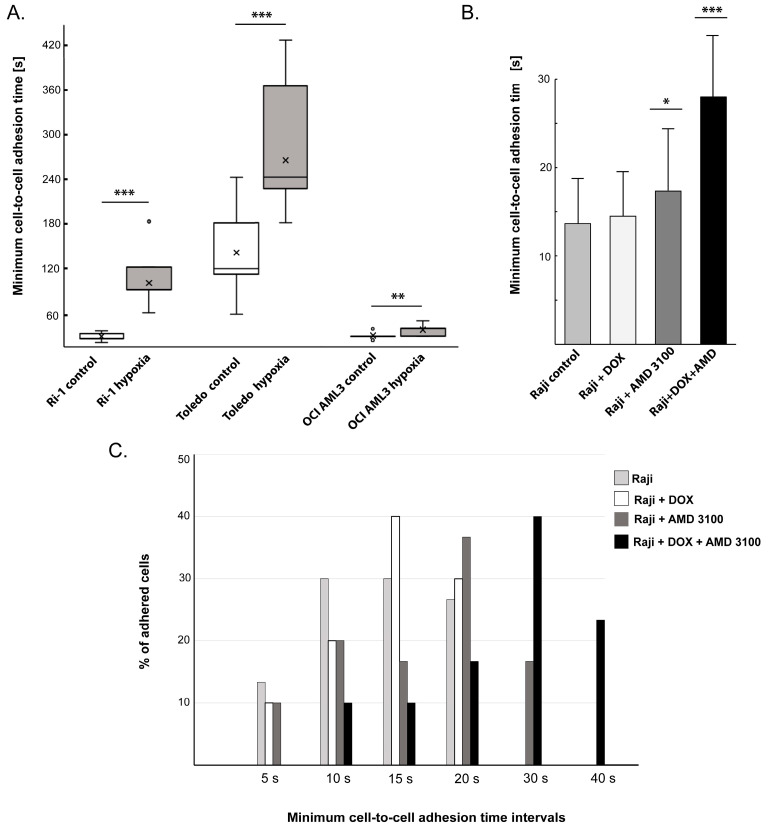
The impact of hypoxia and drug treatment on the minimum cell-to-cell adhesion time of leukemia–lymphoma (LL) cell lines evaluated using optical tweezers (OTs). (**A**) Time-dependent adhesion of LL cell lines to mesenchymal stromal cells (MSCs) under normoxia (21% O_2_) and hypoxia (1% O_2_). (**B**) The effect of anticancer treatment on the adhesive properties of the Raji lymphoma cell line. (**C**) The percentage distribution of drug-treated Raji cells adhered to MSCs within distinct time intervals. DOX—doxorubicin; AMD3100—plerixafor. Statistically significant changes between the drug treatment and control are marked with *, **, and *** for *p* < 0.05, *p* < 0.01, and *p* < 0.001, respectively; Student’s *t*-test. *N* = 30 for each experimental group.

Next, we applied our method to assess changes in cell adhesion in response to hypoxic treatment (Downstream Application 1) and drug exposure (Downstream Application 2). The LL cell lines Ri-1, Toledo, and OCi-AML3 were simultaneously incubated under hypoxia (1% O_2_) and normoxia (21% O_2_). We reported that the difference in adhesive properties between cells exposed to hypoxia and their untreated counterparts remains statistically significant for all tested cell lines (Figure 6A). Moreover, our data highlight the different sensitivities of LL cells to hypoxia, which has been previously documented [52,53,54].

The effect of oxygen deficiency was most pronounced in Ri-1 lymphoma cells, where the minimum cell-to-cell adhesion time increased by 7.7 times, from 12.8 ± 4.9 s to 99 ± 32 s. Incubation in hypoxia had the least effect on the OCI-AML3 leukemic cell line, where the cells formed stable adhesive connections with stromal cells 1.67 times slower than under standard normoxic conditions.

Finally, we used our protocol to determine the minimum cell-to-cell adhesion time after treatment with anti-cancer drugs (Downstream Application 2, Figure 6B,C). The Raji cell line, which represents a very aggressive form of Burkitt lymphoma, was incubated with doxorubicin (DOX), plerixafor (AMD3100), or both drugs for 24 h. Doxorubicin is a cytostatic antibiotic commonly utilized in chemotherapeutic cancer treatments due to its broad anticancer activity, which includes inducing DNA damage, producing reactive oxygen species (ROS), and triggering apoptosis and autophagy [55]. While there are suggestions that DOX influences the activity of cell adhesion molecules (CAMs) involved in adhesion processes, direct evidence supporting this claim is currently lacking. Based on the results obtained using our protocol, untreated Raji cells adhered to stromal cells in 13.7 ± 5.3 s, and treatment with doxorubicin did not significantly affect their adhesive properties compared to the control, as shown in Figure 6B. The incubation of Raji cells with the other tested drug, AMD3100, caused a significant increase in the minimum cell-to-cell adhesion time, suggesting that the drug is associated with a decrease in adhesive properties. In fact, AMD3100 attaches to the CXCR4 receptor, inhibiting its interaction with the natural ligand, stromal-derived factor-1 (SDF-1 or CXCL12). AMD3100 is mainly utilized to improve the mobilization of stem cells for transplantation, especially in cases of hematological malignancies [56]. Importantly, we noticed that combination therapy of DOX with AMD3100 significantly reduced cell adhesion (*p* < 0.001), with a mean minimum cell-to-cell adhesion time of 28 ± 8.72 s.

To summarize, we developed a protocol that allows us to determine the adhesive properties of LL cells on a second time scale. This approach enables us to study the minute changes in lymphocyte adhesion related with disease process, drug treatment, or environmental factors, which are often undetectable by traditional bulk assays.

## 5. Patents

This work is based on the method described in European Patent No. EP3701265: Method for diagnosing neoplasms of lymphoid tissue.

## Figures and Tables

**Table 1:** Seeding density of leukemia–lymphoma (LL) cell lines in T25 culture flask for cell suspension.

Cell Line	Seeding Density (Cells/mL)	Optimal Cell Density in Cell Culture
Ri-1	5 × 10^5^	1–2 × 10^6^
Toledo	5 × 10^5^	1 × 10^6^
Oci-AML3	1–2 × 10^5^	1–2 × 10^6^
Raji	3 × 10^5^	0.5–1 × 10^6^

## Data Availability

The original contributions presented in this study are included in this article/Supplementary Materials. Further inquiries can be directed to the corresponding author.

## Supplementary Materials

The following supporting information can be downloaded at: https://www.mdpi.com/article/10.3390/mps8030059/s1, Video S1: Leukemia–lymphoma cell trapping in optical tweezers; Video S2: Evaluation of minimum cell-to-cell adhesion time in optical tweezers.

## Abbreviations

The following abbreviations are used in this manuscript:

OTs	Optical tweezers
LL	Leukemia–lymphoma
ECM	Extracellular matrix
SC	Stromal cells
BM	Bone marrow
MSCs	Mesenchymal stromal cells
B-NHL	B-cell non-Hodgkin lymphoma
DOX	Doxorubicin
AMD3100	Plerixafor
CAMs	Cell adhesion molecules
SDF-1	Stromal-derived factor-1
FBS	Fetal bovine serum
PBS	Phosphate-buffered saline
DMSO	Dimethyl sulfoxide
ATCC	American Type Culture Collection
DSMZ	German Collection of Microorganisms and Cell Cultures
TB	Trypan blue
NA	Numerical aperture
DM	Dichroic mirror
L	Lens
M	Mirror
OB	Objective
MO	Microscope objective
MS	Microscopic sample
OTr	Optical trap
HIF-1α	Hypoxia-inducible factor 1α
